# A New Method of Colostomy

**Published:** 1911-03-11

**Authors:** 


### SURGERY.

### A NEW METHOD OF COLOSTOMY.

Since inguinal colostomy was popularised some years ago by the late Mr. H. W. Allingham a large number of modifications have been added to his original procedure with the object of rendering the subsequent condition of the patient as bearable as is possible. The degree of success which has attended these efforts to afford the possessor of an artificial anus control over the discharges of his colon is variable, and on the whole not so great as could be wished.

In the *Annals of Surgery* Dr. Howard Lilienthal describes a technique for which he claims a perfect result within a month of operation. Through an incision parallel with and in the outer third of the left rectus he explores the abdomen and draws out all the sigmoid flexure which can be taken out without tension; the upper end of the incision is on a line joining the umbilicus to the anterior iliac spine.

The two legs of the loop of intestine are separated as widely as possible; the upper leg is sutured to the peritoneum and posterior rectus sheath in the upper angle of the wound, and the lower is sutured in a similar manner to the inferior angle. The suture material is silk or linen thread, and the continuous method is used, but every third stitch is tied so as to avoid purse-stringing. The meso-sigmoid in the base of the loop is then sutured through and through to the peritoneum on each side.

The next step is to tie off and divide the gut at the distal end of the loop pulled out. The section is carefully made between a double ligature, and every precaution is taken to avoid any fouling of the tissues from the small edges of exposed mucosa. Pure carbolic acid is applied on a gauze mop for this purpose. The meso-sigmoid is then cut across, and any vessels in it ligatured. Thus at this stage there is a very short distal piece of sigmoid presenting in the lower angle of the wound, and a long proximal piece with attached mesentery in the upper angle. This mesentery is next removed, freeing the long piece of sigmoid entirely, and the whole wound is carefully packed with gauze. If the piece of sigmoid is longer than 3 or 4 inches, the excess should be removed. Four pressure-forceps are then made to seize four equidistant points on the circumference of the cut end, and the gloved finger is passed into the lumen of the gut as far as the place where it is held to the peritoneum of the abdominal wall by suture. An assistant then pulls the clamps round so as to twist the gut on its longitudinal axis from 180 to 360 degrees, according to the thickness of the walls of the sigmoid. When the degree of constriction thus produced is deemed sufficient, as judged by the inserted finger, a few interrupted sutures are passed through the peritoneal coat and submucosa to the aponeurosis of the external oblique, and thus hold the rotated gut in position. It is necessary to make sure by re-examination that a sufficient twist has been accomplished, and if so more sutures are put in to hold the gut firmly to the aponeurosis.

Thus (says the author) a double sphincter is obtained: the first one at the twist, the second, more an angulation than a sphincter, at the point of peritoneal fixation.

The sphincteric action is maintained by the fibres of the rectus as well as by the torsion of the intestine. A few chromic-gut sutures suffice to close the remaining wound in the aponeurosis. A large, rather stiff-walled rubber rectal tube is now inserted about 6 inches into the intestine and tied in place. The remainder of the wound is left open and packed with gauze, and the rubber tube led off into a receptacle at the side of the bed.

About a week after the operation the tube may be withdrawn and any redundant sigmoid burned off with the actual cautery, for which no anæsthesia is required. Often repeated cauterisation is necessary to bring the intestinal mucosa to the skin level during convalescence. Daily irrigation through the tube is desirable to keep the patient's bowels open. The ligature round the lower segment of the bowel is removed in three or four days. If this is not done there is a risk of complete and permanent closure. Potency is necessary to ensure drainage.

The patient gradually learns how to control his colon. He is given a constipating form of diet, and for the first few weeks small doses of deodorised tincture of opium with 20 grains of subgallate of bismuth are administered three to five times a day. By this operation it is said that the frequent discomfort and filthiness of a colostomy opening are almost entirely obviated. The patients have such absolute control that they can even hold a considerable quantity of fluid injected into the colon. The bowels move once or twice a day, and the patient knows when this is about to happen. Further, he is not annoyed by the necessity of wearing an obturator apparatus. Dr. Lilienthal has been performing this operation for eight years, and has followed up the after-careers of many of those for whom he has done it.

